# Predictors of mortality among TB-HIV co-infected children attending anti-retroviral therapy clinics of selected public hospitals in southern, Ethiopia: retrospective cohort study

**DOI:** 10.1186/s13690-021-00713-1

**Published:** 2022-01-04

**Authors:** Jifare Gemechu, Bereket Gebremichael, Tewodros Tesfaye, Alula Seyum, Desta Erkalo

**Affiliations:** 1 College of Medicine and Health Sciences, Wachemo University, Hosanna, Ethiopia; 2 College Health Sciences, Addis Ababa University, Addis Ababa, Ethiopia

**Keywords:** TB-HIV/AIDS, Mortality, Children, Anti-retroviral therapy

## Abstract

**Background:**

Co-infection of tuberculosis and HIV has a significant impact on public health. TB is the most common opportunistic infection and the leading cause of death in HIV-positive children worldwide. But there is paucity of studies concerning the predictors of mortality among TB-HIV co-infected children. This study aimed to determine the predictors of mortality among TB-HIV co-infected children attending ART clinics of public hospitals in Southern Nation, Nationalities and Peoples Region (SNNPR), Ethiopia.

**Methods:**

A hospital-based retrospective cohort study design was used among 284 TB-HIV co-infected children attending ART clinics at selected public hospitals in SNNPR, Ethiopia, from January 2009 to December 2019. Then, medical records of children who were TB/HIV co-infected and on ART were reviewed using a structured data extraction tool. Data were entered using Epidata 4.6 and analyzed using SPSS version 23. The Kaplan Meier survival curve along with log rank tests was used to estimate and compare survival time. Bivariable and multivariable analyses were conducted to identify predictors of mortality among TB/HIV co-infected children. Adjusted Hazard Ratio with *p* value < 0.05 and 95% confidence interval was considered statistically significant.

**Result:**

A total of 284 TB/HIV co-infected children were included in the study. Among these, 35 (12.3%) of them died during the study period. The overall mortality rate was 2.78 (95%CI = 1.98-3.99) per 100 child years of observation. The predictors of mortality were anemia (AHR = 3.6; 95%CI: 1.39-9.31), fair or poor ART drug adherence (AHR = 2.9; 95%CI = 1.15-7.43), extrapulmonary TB **(**AHR = 3.9; 95%CI: 1.34-11.45) and TB drug resistance (AHR = 5.7; 95%CI: 2.07-15.96).

**Conclusion:**

Mortality rate of TB/HIV co-infected children in selected public hospitals in SNNPR, Ethiopia was documented as 2.78 per child years of observation as a result of this study. Moreover, Anemia, drug resistant tuberculosis, extrapulmonary TB and poor adherence to ART drugs were identified as the predictors of mortality among these children.

## Background

Tuberculosis (TB) is a chronic communicable bacterial infection caused primarily by *Mycobacterium tuberculosis* and occasionally by *Mycobacterium africanum*, Mycobacterium canetti, or *Mycobacterium bovis* [1]. It mainly affects the lungs (pulmonary TB), but it can sometimes affect other sites, which is known as extrapulmonary TB. It is spread through inhalation of mucus droplets when an infected person coughs or sneezes and releases bacteria into the air. Usually, the clinical manifestation in children is non-specific. However, the most common symptoms include a non-productive cough, a fever lasting more than 2 weeks, weight loss, night sweats, loss of appetite, and decreased activity. Whereas the symptom of extrapulmonary TB is dependent on the site affected [2].

People with compromised immune systems, such as those suffering from human immunodeficiency virus (HIV), malnutrition, or diabetes mellitus (DM), are at a higher risk of developing the disease. Almost all HIV-positive people and 40% of HIV-negative people with tuberculosis who do not receive effective treatment will die [3]. HIV destroys and damages immune cells, resulting in immunodeficiency. This immunodeficiency increases susceptibility to diseases such as tuberculosis [3, 4].

TB/HIV co-infection poses a challenge to human survival because of the mutual impact. HIV complicates each aspect of TB including presentation, diagnosis, and treatment5. As the viral load rises, tuberculosis speeds up the progression of HIV infection to AIDS and consequently, leads to death. On the other hand, HIV infection reduces the CD4 count leading to depressed immunity through which it enhances the activation of latent TB and increases the relapse rate of TB. Their combination increases the burden on the health system, the development of extrapulmonary TB and negative sputum smears [1].

Treatment of HIV/TB co-infected patients may result in difficulties such as drug interactions, toxicity, immunological reconstitution inflammatory syndrome (IRIS), and decreasing plasma drug level, leading to drug resistance treatment regardless of adherence. TB may potentially be over diagnosed in HIV-infected children due to the presence of various illnesses that mimic TB [2]. The diagnosis of tuberculosis in HIV-infected children may be problematic due to the absence of tuberculin skin test reactivity, the difficulties of culture confirmation, and the clinical similarities of TB to many other HIV-related infections [5].

The disease burden obviously varies among countries, ranging from less than five to more than 500 new cases per 100,000 population per year, with a global average of 130. The majority of the TB cases in the aforementioned study occurred in the WHO regions of Asia, Africa, and the Western Pacific, accounting for 44, 24, and 18%, respectively [6, 7]. According to the 2018 report of the joint United Nations program on HIV and AIDS (UNAIDS), 37.9 million people worldwide are infected with HIV. Among these, 1.7 million are children < 15 years and estimated 84,000 newly infected children are living in eastern and southern Africa [8].

In 2018, people living with HIV accounted for an estimated 8.6% of all incident TB cases. The proportion of TB/HIV co-infection cases was highest in World Health Organization (WHO) African Region nations, topping 50% in portions of southern Africa [6]. According to the Ethiopia Population-based HIV Impact Assessment (EPHIA), the prevalence of HIV among children aged 0-14 in urban Ethiopia in 2018 was 0.3% [9].

According to WHO, the prevalence of TB in Ethiopia in 2018 was estimated to be 151/100,000, with a mortality rate of 2% ranging from 1.4 to 2.8% among HIV-positive people [6, 3]. According to WHO, one out of every three HIV-related deaths is caused by tuberculosis [10]. It is also the most common opportunistic infection in HIV-positive children, and it is still one of Ethiopia’s top ten causes of death [11].

Several comprehensive programs have been designed and implemented to prevent, diagnose, and treat TB among HIV positives. However, the problem remains a major global and national health concern that requires extensive work to reach the SDG and end TB. Even though there have been studies on predictors of mortality in TB/HIV co-infected adults, there have been no previous sufficient scientific investigations done on the literature in the pediatric age range, which did not cover some characteristics such as TB medication resistance. Therefore, this study will bridge this information gap and update the previous knowledge on the same problem.

## Methods and materials

### Study design, setting and period

A hospital-based retrospective cohort study design was used in ART clinics at selected public hospitals in SNNPR, Ethiopia. The study was conducted from February to March 2020, and the 284 TB-HIV co-infected children cohort was followed from January 2009 to December 2019 at ART clinics of selected public hospitals in SNNPR. SNNPR is an administrative region in Ethiopia. It is Ethiopia’s third-largest administrative region, with nine administrative regions. In terms of culture, language, and ethnicity, it is also the most diverse region in the country. It is divided into 14 zones, one city administration, and four special woredas. Hawassa is its capital city, located 273 km from Addis Ababa, Ethiopia’s capital city. According to the 2007 census, the region has an estimated population of 18.9 million people. The region has four referral hospitals and forty general hospitals. The study included six hospitals: three referral (Hawasa University Referral Hospital, Wolaita Sodo University Teaching Referral Hospital, and Nigist Eleni Mohammad Memorial Referral Hospital) and three general (Yirgalem, Arba Minch, and Jinka).

### Sample size determination

The sample size was calculated by using Epi info version 7 statistical packages for cohort study, by considering 95% CI and a power of 80%, from a study conducted in Gondar, using CPT and IPT as the key predictor factors [12]. As a result the parameters: IPT (percent of exposed with the outcome P1 = 18.4%, percent of non-exposed with the outcome P2 = 6.2%) was used as the independent predictor since it gives the maximum sample size. r: is the non-exposed to exposed ratio of 1:1. The largest sample size (*N* = 288) was then chosen as the final sample size for the investigation.

### Sampling procedure

The study was conducted at selected public hospitals in SNNPR. Purposive sampling was used to select the hospitals with high patient volume. Then, medical record numbers of children who are TB/HIV co-infected and on ART from January 1st, 2009 to December 31, 2019 were identified. The ART log book was used to look for those with history of tuberculosis. Six hospitals were included in the study. The survey identified 325 children in the ART log book at Hawasa University Referral Hospital (65), Wolaita Sodo University Teaching Referral Hospital (61), Nigist Eleni Mohammad Memorial Referral Hospital (57), Yirgalem General Hospital (40), Arba Minch General Hospital (57), and Jinka General Hospital (45). From the 325 identified charts, 284 were allocated proportionally to the study. The rest of the charts were excluded due to incompleteness.

### Measurement of terms

**ART adherence-** classified as Good (≥95% or Miss≤3 doses per month), Fair (85-94% or Miss 4-8 doses), and Poor (< 85% or Miss> 9 doses) according to calculated percentage of ART drug taken from the total monthly dosage.

**Loss to Follow Up-** When the child misses appointment for more than 3 months.

**Transferred Out-** when a child is transferred to other health facility.

**Treatment Failure-** classified by clinical criteria, immunologic criteria and virologic criteria.

**Clinical Treatment Failure** is the detection of new or recurrent WHO clinical stage of III or IV.

**Immunologic Treatment Failure** is a drop in CD4 level, after the initial immune recovery next to ART initiation to values at or below the age-related CD4 threshold for treatment initiation OR 30% drop of CD4% or value from highest levels after therapy.

**Virologic Failure -persistent** viral load exceeding 5000 copies/ml in a child who is fully adhered to treatment and who was on ART for at least 24 weeks.

**Time to death**- the time between the diagnosis of TB/HIV co-infection and occurrence of the event (death) during the follow up period.

**Censored**- If the child is loss to follow up or transferred out before developing the event or if the child is alive at the end of the study period.

**Drug resistant TB;** classified in to Multi drug resistant (MDR), resistance to isoniazid and rifampicin, and Extensive drug resistant (XDR). XDR- resistance to at least four core anti TB drugs involving isoniazid, rifampicin, any of the fluroquinolons like moxifloxacin or levofloxacin and to at least one of the second line drugs which are amicacin, capreomaycin or kanamycin.

**Immune suppression:** classified as severe, advanced, and none or not significant based on WHO classification as shown in Table 1.

**Table 1:** Immune suppression-Based on WHO classification

HIV-associated immunodeficiency	Age-related CD4+ values			
	< 11 months (%CD4+)	12–35 months (%CD4+)	36–59 months (%CD4+)	> 5 years (absolute number per mm3 or %CD4+)
None or not significant	> 35	> 30	> 25	> 500
Mild	30–35	25–30	20–25	350 − 499
Advanced	25–29	20–24	15 − 19	200 − 349
Severe	< 25	< 20	< 15	< 200 or < 15%

### Data collection procedure

A data extraction tool was developed from the national ART entry and follow up form that is currently used by the ART clinics of the study area. The data were collected by six BSc and five diploma nurses working in the ART clinic of respective hospitals. They used the data collection tool to extract the information from children’s charts. Charts were collected by using the children’s registration number which is found in the data base in the computer system. In addition, data clerks helped in identifying the charts.

### Data quality control

To maintain the quality of the data, data extraction tool was carefully adapted from the national follow-up care forms with some modification. One-day training was given regarding the objectives and variables of the study and how to extract data by using the tool was given. Moreover, a periodic supervision took place. In addition, the data extraction tool was pretested to check the consistency.

### Data processing and analysis

Before data entry, all questionnaires were checked for completeness. Cleaned and coded data was entered into Epi Data version 4.6 and analysis of the data was conducted by using the statistical package for social sciences (SPSS) version 23. Cox proportional hazard model assumption was checked using Schoenfeld, residual test. Patients’ cohort characteristics for continuous data were described in terms of central tendency (mean or median) and dispersion (standard deviation or inter quartile range). Frequency distribution was used for categorical data. The Kaplan Meier survival curve was used to estimate and compare survival time and log rank tests was used to compare survival curves. Bivariate Cox-proportional hazards regression model was fitted for each explanatory variables and multivariable Cox model was used to identify predictors of mortality among TB/HIV co-infected children. Adjusted Hazard Ratio with its 95% confidence interval was used to estimate the strength of association and *p* value ≤0.05 was considered as statistically significant. Proportional hazard assumption was tested and satisfied.

## Results

### Socio demographic characteristics

A total of 325 charts of children on ART and had a history of tuberculosis were reviewed. However, 41 charts were excluded from analysis due to incomplete data. Therefore, 284 charts of children with TB/HIV co-infection were included in the analysis. Among these, almost half (48.2%) were male with male to female ratio of 1: 1.07 and most of the children (47.9%) were among the age group of 6-10 years. The mean age of the study participants was 7.1(SD ± 3.7) years. Almost one-third (74.6%) of the children’s care givers were HIV positive. In addition, most of the care givers that is, 133(46.8%) were among the age group of 25-34 years (Table 2).

**Table 2:** Socio-demographic characteristics of TB/HIV co-infected children attending ART clinics of selected public hospitals in SNNPR, Ethiopia, from January 2009 to December 2019

Characteristics		Total	Censored	Death	Log Rank test	
					X^**2**^	***P***- value
**Age**	< 1	18 (6.3)	17 (94.4)	1 (5.6)	1.79	0.61
	1-5	78 (27.5)	68 (87.2)	10 (12.8)		
	6-10	136 (47.9)	116 (85.3)	20 (14.7)		
	11-15	52 (18.3)	48 (92.3)	4 (7.7)		
**Sex**	Male	137 (48.2)	121 (87.1)	19 (12.9)	0.00	0.95
	Female	147 (51.8)	128 (88.3)	16 (11.7)		
**Age of care giver**	15-24	40 (14.1)	36 (90)	4 (10)	2.70	0.44
	25-34	133 (46.8)	114 (85.7)	19 (14.3)		
	35-44	82 (28.9)	74 (90.2)	8 (9.8)		
	> 44	29 (10.2)	25 (86.2)	4 (13.8)		
**Child lives with**	Parents	239 (84.2)	210 (97.9)	29 (12.1)	5.46	0.14
	Guardian	17 (6)	16 (94.1)	1 (5.9)		
	Orphan	9 (3.2)	6 (66.7)	3 (33.3)		
	Others	19 (6.7)	17 (89.5)	2 (10.5)		
**Child care giver**	Mother	186 (65.5)	162 (87.1)	24 (12.9)	2.85	0.58
	Father	35 (12.3)	30 (85.7)	5 (14.3)		
	Step parent	11 (3.9)	10 (90.9)	1 (9.1)		
	Sibling	17 (6)	17 (100)	0 (0)		
	Others	35 (12.3)	30 (85.7)	5 (14.7)		
**Mother HIV status**	Positive	223 (77.8)	191 (85.7)	32 (14.3)	3.30	0.19
	Negative	3 (1.1)	3 (100)	0 (0)		
	Unknown	58 (20.4)	55 (94.8)	3 (5.2)		
**Care giver HIV status**	Positive	212 (74.6)	183 (86.3)	29 (13.7)	2.15	0.34
	Negative	28 (9.9)	27 (96.4)	1 (3.6)		
	Unknown	44 (15.5)	39 (88.6)	5 (11.4)		

### Child clinical characteristics

From the 284 participants, majority of them (91.9%) had opportunistic infection at baseline. Out of this, pulmonary TB contributes the highest proportion which is, 63% followed by pneumonia (17.3%) and diarrhea (9.9%). Most (41.2%) of the children were eligible for HAART by WHO stage criteria. Forty (14.1%) of the children had treatment failure. Virologic failure taking the highest proportion [13] followed by immunologic failure [11] and clinical failure [3] but only 24 of them were on second line ART drug. On the other hand, 14.8% of the children had a history of previous TB. Concerning ART drug adherence, 93.7% of the participants had good adherence in the first 3 months of ART initiation (Table 7).

**Table 3:** Clinical characteristics of TB/HIV co-infected children attending ART clinics of selected public hospitals in SNNPR, Ethiopia, from January 2009 to December 2019

Clinical characteristics		Total	Censored	Dead	Log Rank test	
					X^**2**^	***P***- value
Baseline WHO stage	I & II	60 (21.1)	143 (96.6)	5 (3.4)	1.09	0.29
	III & IV	224 (78.9)	106 (77.9)	30 (22.1)		
ART eligibility criteria	CD4+ cells	12 (4.2)	12 (100)	0 (0)	14.35	0.001
	WHO stage	117 (41.2)	109 (93.2)	8 (6.8)		
	Both	78 (27.5)	62 (79.5)	16 (20.5)		
	Not recorded	77 (27.1)	66 (85.7)	11 (14.3)		
Baseline Opportunistic infection	Yes	261 (91.9)	229 (87.7)	32 (12.3)	0.44	0.50
	No	23 (8.1)	20 (87)	3 (13)		
Initial regimen	D4T based	147 (51.8)	129 (87.8)	18 (12.2)	0.66	0.88
	AZT based	104 (36.6)	90 (86.5)	14 (13.5)		
	TDF based	16 (5.6)	15 (93.8)	1 (6.2)		
	ABC based & 1j	17 (6)	15 (88.2)	2 (11.8)		
Initial regimen change	Yes	135 (47.5)	121 (89.6)	14 (10.4)	1.87	0.17
	No	149 (52.5)	128 (85.9)	21 (14.1)		
Reason for regimen change	Side effect	33 (11.6)	29 (87.9)	4 (12.1)	2.6	0.62
	Treatment failure	24 (8.5)	20 (83.3)	4 (16.7)		
	TB	30 (10.6)	27 (90)	3 (10)		
	Stock out	35 (12.3)	32 (91.4)	3 (8.6)		
Treatment failure	Yes	40 (14.1)	37 (92.5)	3 (7.5)	1.71	0.19
	No	244 (85.9)	212 (86.9)	32 (13.5)		
Baseline HIV related immune suppression	Mild/non-significant	172 (60.6)	164 (95.3)	8 (4.7)	33.24	0.001
	Advanced	46 (16.2)	40 (87)	6 (13)		
	Severe	66 (23.2)	45 (68.2)	21 (31.8)		
INH	Yes	182 (64.1)	163 (89.6)	19 (10.4)	2.24	0.13
	No	102 (35.9)	86 (84.3)	16 (15.7)		
CPT	Yes	260 (91.5)	227 (87.3)	33 (12.3)	0.05	0.81
	No	24 (8.5)	22 (91.7)	2 (8.3)		
Baseline hemoglobin	≤10	104 (36.6)	89 (76.7)	27 (23.3)	21.05	0.001
	> 10	180 (63.4)	249 (95.2)	35 (12.3)		
Adherence	Good	266 (93.7)	238 (89.5)	28 (10.5)	12.71	0.001
	Fair/poor	18 (6.4)	11 (61.1)	7 (38.9)		
Site of TB	Pulmonary	218 (76.8)	232 (90.3)	25 (9.7)	17.59	0.001
	Extrapulmonary	66 (23.2)	17 (63)	10 (33)		
Time of TB diagnosis	Pre ART	171 (60.3)	153 (89.5)	18 (10.5)	3.65	0.55
	ART	113 (39.8)	96 (85)	17 (15)		
Previous history of TB	Yes	42 (14.8)	29 (69)	13 (31)	19.85	0.001
	No	242 (85.2)	220 (90.9)	22 (9.1)		
TB drug resistance	No	264 (93)	237 (89.8)	27 (10.2)	20.29	0.001
	MDR	20 (7)	12 (60)	8 (40)		

### Mortality rate

**Fig. 1:**
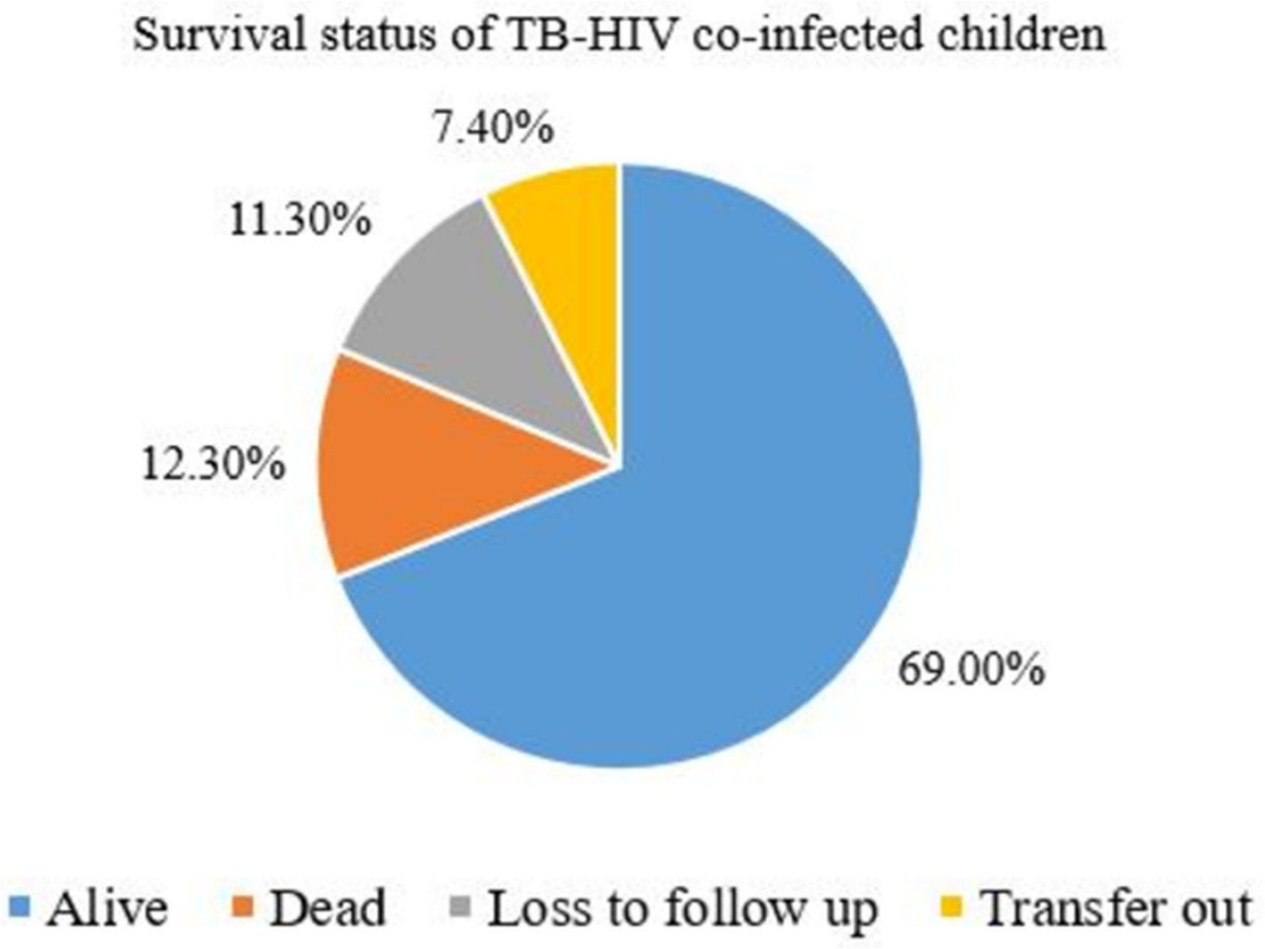
The survival status of TB-HIV co-infected children attending ART clinics of selected public hospitals in SNNPR, Ethiopia, from January 2009 to December 2019

Out of the 284 children in this study, 196 (69%) were alive, 35 (12.3%) died, and 32 (11.3%) were lost to follow-up (Fig. 1). 284 children with TB/HIV co-infection and on ART follow up were followed for different periods of time with total of 1257.17 child-years of observation. They were followed for a minimum of 1 month to a maximum of 10 years. The median follow up time was 4.5 years. A total of 35(12.3%) deaths was observed through the follow up time making the overall mortality rate to be 2.78 per 100 person year of follow up (95%CI = 1.98-3.99) for the study cohort. From those who died, 16(45.78%) of them were male, 60% of them were WHO stage 4, 65.7% of them had CD4 level less than two hundred.

The mortality rate of children with extrapulmonary tuberculosis is higher than those with pulmonary Tuberculosis which is 9.6 per 100 child years compared to 2.16 per 100 child years. Moreover, the mortality rate of children with MDR TB (12.53 per 100 child-years) is much higher than those without drug resistant TB (2.26 per 100 child- year) of follow up (Table 4).

**Table 4:** Mortality rate stratified by selected socio demographic and clinical characteristics of TB/HIV co-infected children

Characteristics		Total	N PY	Death	N IDR
Age	≤1	18 (6.3)	75.66	1	1.32
	1-5	78 (27.5)	426.33	10	2.34
	6-10	136 (47.9)	590.58	20	3.38
	11-15	52 (18.3)	164.58	4	2.43
Mother HIV status	Positive	219 (77.1)	1028.08	32	3.11
	Negative/unknown	65 (22.9)	229.08	3	1.3
Baseline WHO stage	I & II	60 (21.1)	212	4	1.88
	III & IV	224 (78.9)	1045.166	31	2.96
Baseline OI	Yes	261 (91.9)	1200	32	2.67
	No	23 (8.1)	57.16	3	5.24
Baseline HIV related immune suppression	Non-significant/mild	172 (65.9)	800.167	8	9.99
	Advanced	46 (16.1)	196.75	6	3.04
	Sever	66 (18.1)	260.25	21	8.06
Hemoglobin level	≤10	104 (36.6)	455.91	27	5.92
	> 10	180 (63.4)	801.2	8	0.99
Treatment Failure	Yes	40 (14.1)	222	3	1.35
	No	244 (85.9)	1035	32	3.09
IPT	Yes	182 (64.1)	874	19	2.17
	No	102 (35.9)	383.16	16	4.17
CPT	Yes	260 (91.5)	11.83	33	2.8
	No	24 (8.5)	81.3375	2	2.45
Adherence	Good	266 (93.7)	1193.33	28	2.36
	Fair/poor	18 (6.4)	63.83	7	10.96
Site of TB	Pulmonary	218 (76.8)	1153.66	25	2.16
	Extrapulmonary	66 (23.2)	103.5	10	9.66
TB drug Resistance	No	264 (93)	1193.33	27	2.26
	MDR	20 (7)	63.83	8	12.53

*PY* person year, *IDR* incidence density rate.

### Predictors of mortality

**Fig. 2:**
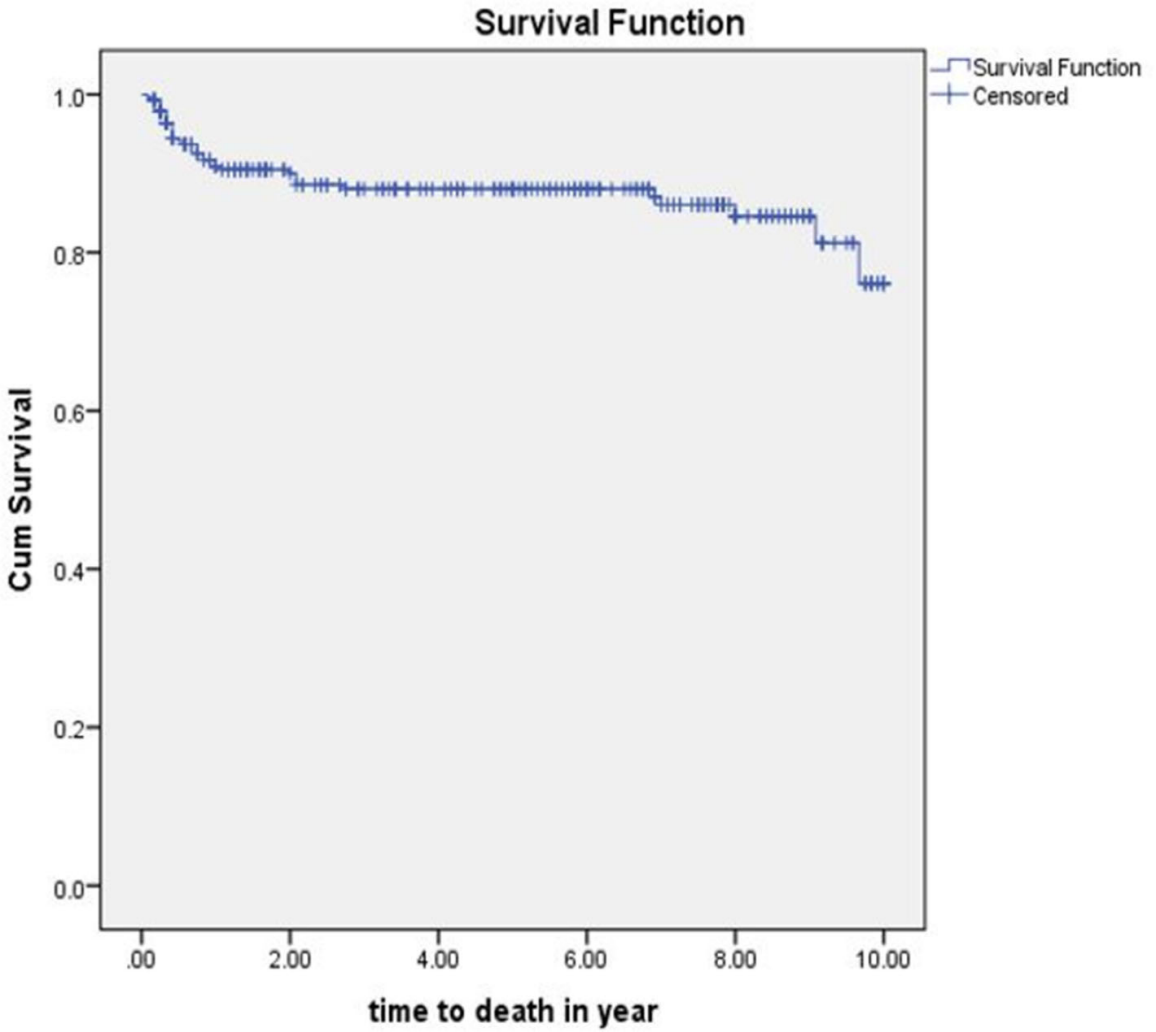
Kaplan-Meier curve of survival proportion for TB/HIV co-infected children attending ART clinics of selected public hospitals in SNNPR, Ethiopia, from January 2009 to December 2019

The equality of survival for different categories of explanatory variables was tested by Log rank (Mantel-Cox) test. Hemoglobin level, ART drug adherence, site of TB and TB drug resistance were significantly associated with time to death. The mean survival time of the whole follow-up was 8.8 (95%CI; 8.4-9.15) years (Fig. 2).

**Fig. 3:**
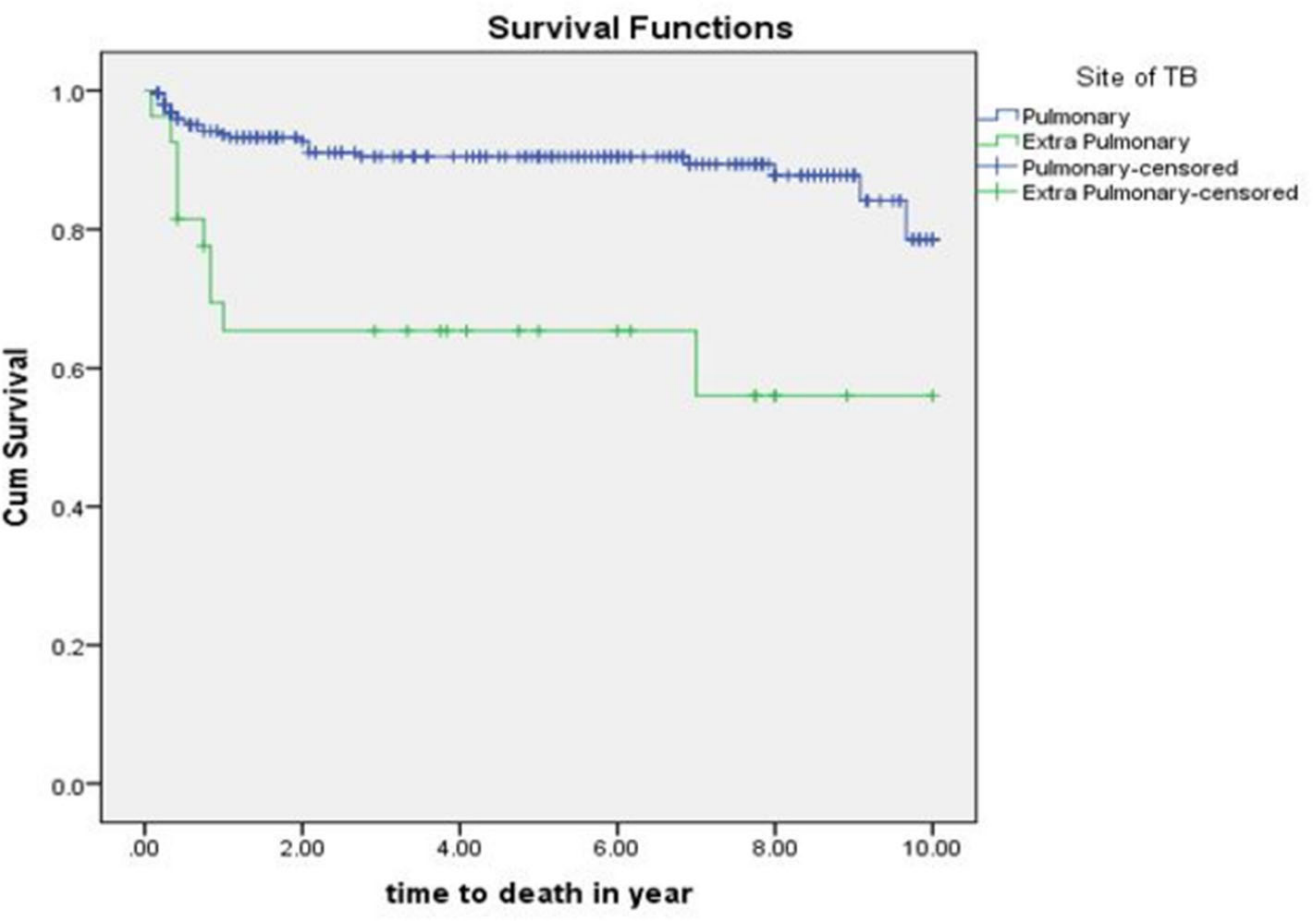
The Kaplan- Meier estimates of survival function by site of TB infection of TB/HIV co-infected children attending ART clinics of selected public hospitals in SNNPR, Ethiopia from January 2009 to December 2019

The mean survival time for those with extrapulmonary tuberculosis was 6.5(95%CI: 4.7-8.2) years whereas it was 9(95%CI: 8.7-9.4) years for those with pulmonary tuberculosis with significance level of *p* = 0.0001 (Fig. 3).

**Fig. 4:**
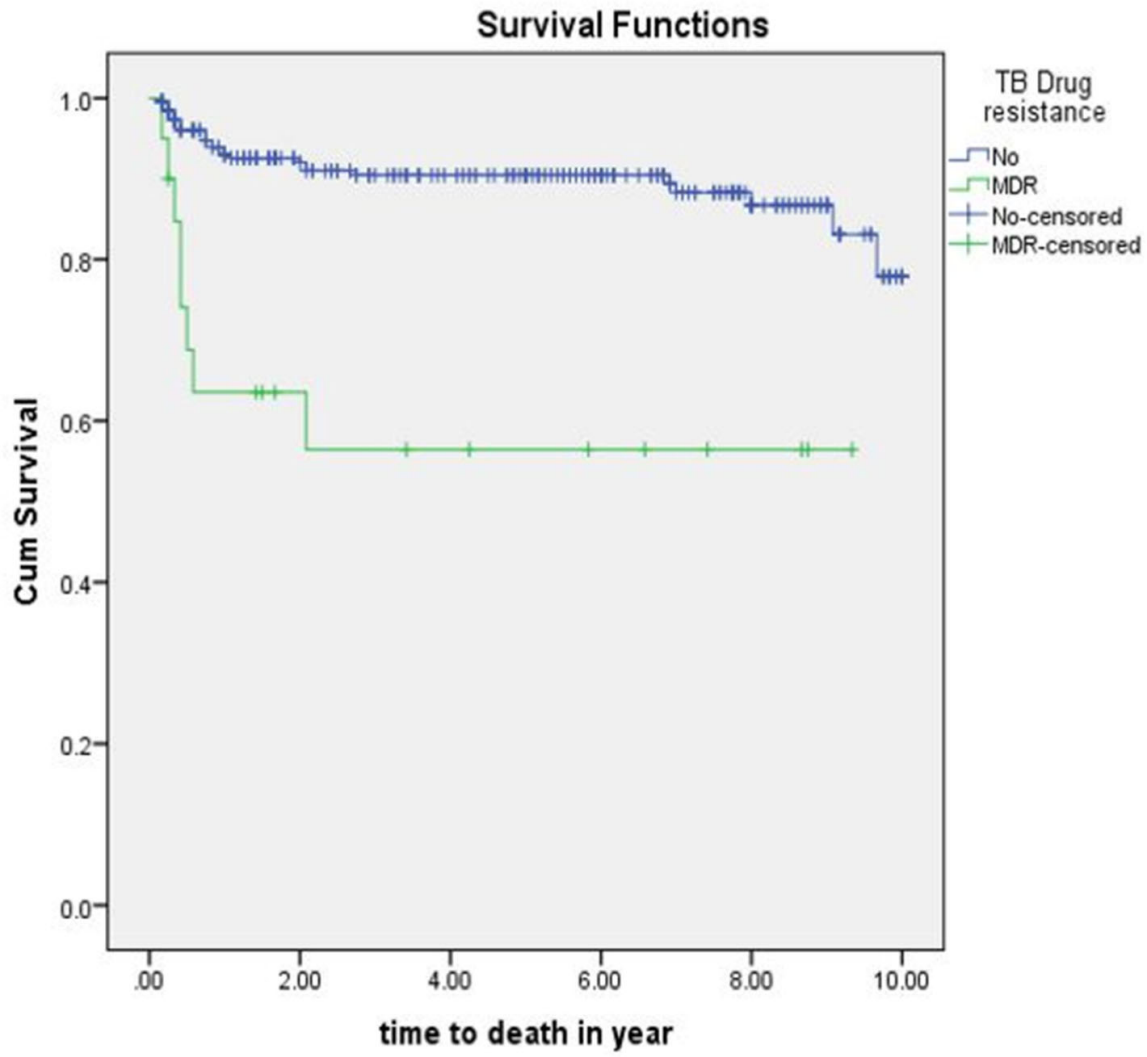
The Kaplan- Meier estimates of survival function by TB drug resistance status of TB/HIV co-infected children attending ART clinics of selected public hospitals in SNNPR, Ethiopia from January 2009 to December 2019

The mean survival time of those with multi drug resistant tuberculosis and HIV co-infected is lower 5.5 (95%CI: 3.5-7.5) years when compared with those with no drug resistances 8.9(95%CI: 8.6-9.3) years (*p* = 0.001) (Fig. 4).

**Fig. 5:**
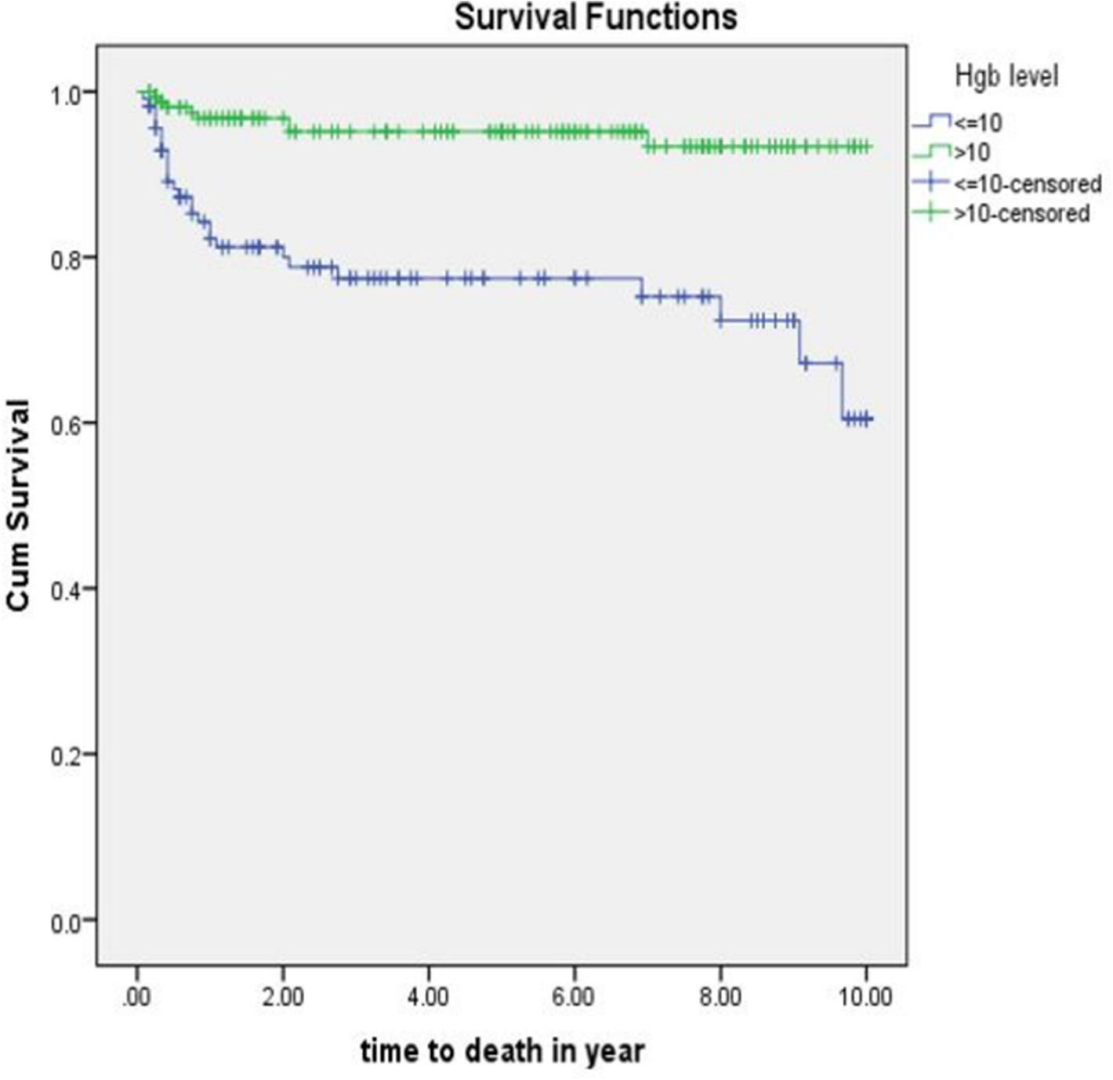
Kaplan –Meier survival curve of hemoglobin level of TB/HIV co-infected children attending ART clinics of selected public hospitals in SNNPR, Ethiopia from January 2009 to December 2019

The mean survival time for children with hemoglobin level ≤ 10 g/dl was 7.73(95%CI: 6.98-8.48) years where as for those who had hemoglobin level above 10 g/dl was 9.51(95%CI: 9.18-9.84) years (*p* value = 0.001) (Fig. 5).

**Fig. 6:**
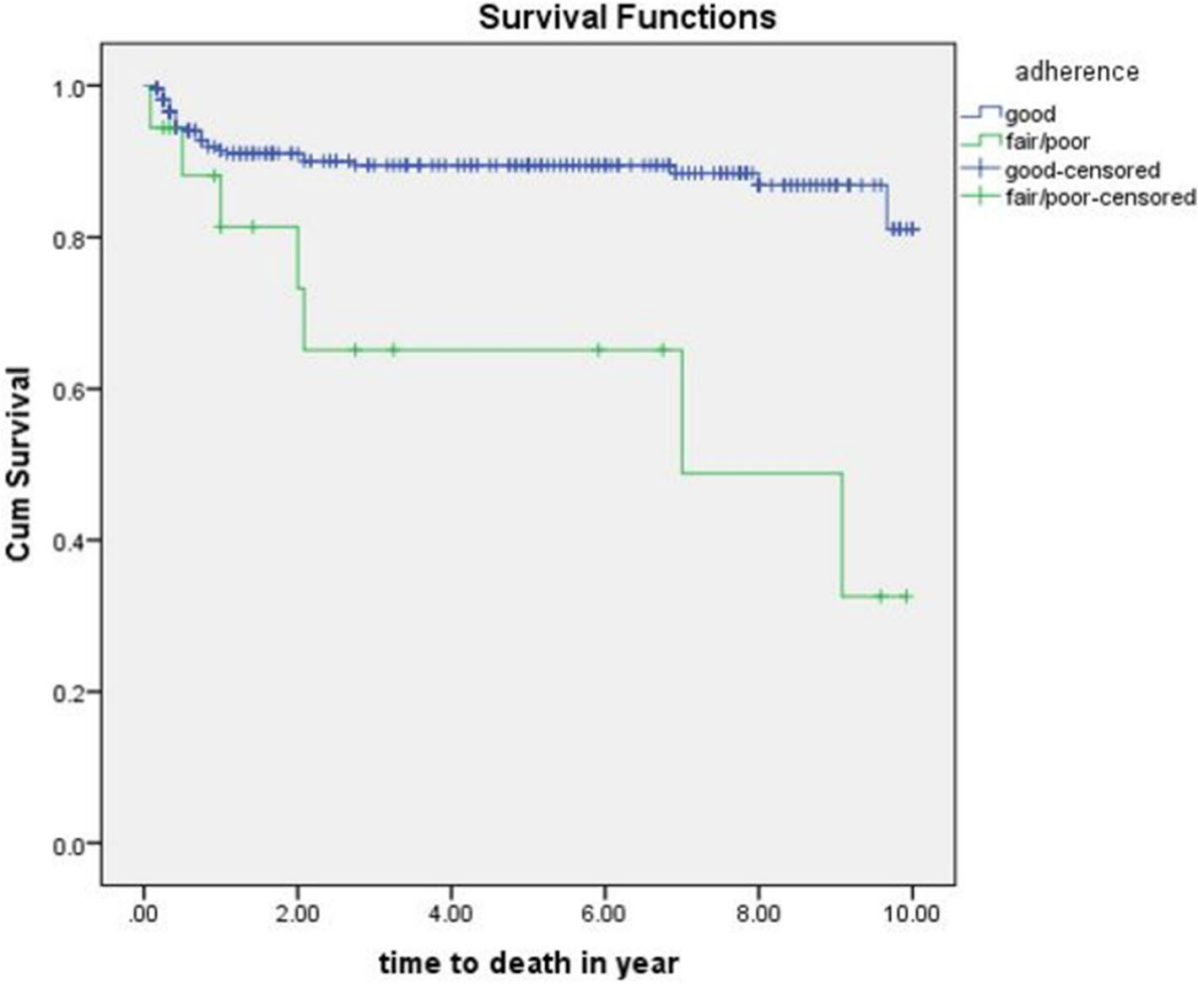
The Kaplan- Meier estimates of survival function by ART drug adherence of TB/HIV co-infected children attending ART clinics of selected public hospitals in SNNPR, Ethiopia from January 2009 to December 2019

Individuals who have good ART drug adherence have greater mean survival time than who had fair or poor adherence which was 8.95 (95%CI: 8.52-9.31) years compared to 6.28 (95%CI: 4.17-8.36) years (*p* value = 0.001) (Fig. 6).

### Predictors of mortality among TB/HIV co-infected children

The bivariable Cox regression analysis showed that ART drug adherence, site of TB, previous history of TB, time of TB diagnosis, WHO stage at TB, drug resistant TB, Hemoglobin level at baseline, Isoniazid prophylaxis (IPT), presence of Pulmonary TB, extrapulmonary TB, oral and esophageal thrush at baseline, and adherence to ARV drugs were associated with mortality of TB/HIV co-infected children. But site of TB, drug resistant TB, hemoglobin level and ART drug adherence remained statistically significant predictors of mortality among TB/HIV co-infected children in multivariable Cox-regression analysis.

According to this study, the hazard of dying for TB/HIV co-infected children with hemoglobin level of < 10 g/dl was higher than those with hemoglobin level of > 10 g/dl (AHR = 3.6; 95%CI: 1.39-9.31). Individuals who had drug resistant TB have 5.7 times higher hazard of dying than those who had no drug resistant TB (AHR = 5.7;95%CI: 2.07-15.96). Children who had good adherence had 2.9 times higher hazard of dying than those with fair or poor adherence (AHR = 2.9; 95%CI = 1.15-7.43). Moreover, those children with extrapulmonary TB have 3.9 times higher hazard of dying than those with pulmonary TB **(**AHR = 3.9; 95%CI: 1.34-11.45) (Table 5).

**Table 5:** Cox-regression analysis of predictors of mortality of TB/HIV co-infected children attending ART clinics of public hospitals in SNNPR, Ethiopia from January 2009 to December 2019

Variables	Categories	Death	Censored	CHR	AHR
Time of TB diagnosis	Pre ART	18	153	0.52 (0.26-1.03)	0.5 (0.18-1.36)
	ART	17	96	1	1
WHO stage at TB diagnosis	III	14	169	1	1
	IV	21	80	3.0 (1.53-5.95)	1.61 (0.71-3.67)
Hemoglobin level	≤10 mg/dl	27	89	5.23 (2.37-11.54)	3.6 (1.39-9.31)*
	> 10 mg/dl	8	160	1	1
Site of TB	Pulmonary	25	232	1	1
	Extrapulmonary	10	17	4.2 (2.02-8.81)	3.9 (1.34-11.45)*
TB drug resistance	No	27	237	1	1
	MDR	8	12	5.1 (2.31-11.31)	5.7 (2.07-15.96)*
ART drug adherence	Good	28	238	1	1
	Fair/Poor	7	11	4.02 (1.75-9.22)	2.9 (1.15-7.43)*
Previous history of TB	Yes	14	53	4.1 (2-8.33)	2.63 (1.07-6.44)
	No	21	196	1	1
ART treatment failure	Yes	3	37	0.46 (0.14-1.5)	0.56 (0.16-1.93)
	No	212	32	1	1
IPT	Yes	19	163	1	1
	No	16	86	1.65 (0.85-3.22)	0.55 (0.26-1.15)
Baseline PTB	Yes	20	159	0.55 (0.27-1.14)	2.3 (0.66-8.17)
	No	15	90	1	1
Baseline EPTB	Yes	4	13	2.3 (0.8-6.5)	0.97 (0.24-3.9)
	No	31	236	1	1
Baseline oral thrush	Yes	3	11	2.44 (0.74-8.01)	3.7 (1.02-13.39)
	No	32	238	1	1
Baseline esophageal thrush	Yes	2	6	3.3 (0.79-13.93)	4.4 (0.77-25.02)
	No	33	243	1	1

*CHR* crude hazard ratio, *AHR* adjusted hazard ratio, *significantly associated variables with *P* < 0.05.

## Discussion

The mortality rate of children with TB/HIV co-infection was 2.78 per 100 child years of follow up and the proportion of mortality was 12.3% within the study population. The rate of mortality is slightly lower than a cohort study conducted in Gondar [12]. The reason for this might be because the previous study was conducted over twelve years of period which is longer than the study period of this study. Moreover, this finding is lower than cohort study conducted in South Africa [13]. This may be explained by the fact that the previous study was conducted only on children younger than 2 years of age. This age group is high risk group because of immature immune system leading to severe disease and death.

Additionally, according to the study, one third of the study participants had TB before ART was initiated for them. This may also contribute to death because the presence of TB may further weaken the immune system leading to increased viral load which results in severe immune suppression. On the other hand, this result is a little higher than a study conducted in Nigeria [14]. This may be explained by the previous study did not include children who were co-infected with extrapulmonary tuberculosis. These children are at a greater risk of mortality due to the severity of the disease. Therefore, the inclusion of extrapulmonary TB and HIV co-infected children in this study might be the reason for higher mortality rate.

The proportion of mortality in this study which is 12.3% is concordant to the studies done in Tanzania [15], Nigeria [16], Malawi [17], South Africa [18], meta-analysis in South Africa [19] and Uganda [20]. Furthermore, only slight difference from an observational cohort study conducted in four countries [21] and South Africa [22]. This may be due to the similarities in the burden of HIV, TB and HIV/TB co-infection as all study areas are classified under high burden countries and the similarities in the age group of study participants may be another reason. However, the finding was lower from studies done in Thailand [23], India [24], and Congo [25]. This difference may be due to the difference in number of study participants from the study in Thailand. Furthermore, the studies from India and South Africa were conducted only on MDR TB/HIV co-infected children who are at high risk of mortality.

According to this study, the overall cumulative survival time of the study period was 76.1% which is comparable with study conducted in Gondar [12]. This may be due to the similarities in study participant’s age, socio demographic characteristics and management protocols of TB, HIV and TB/HIV co-infection. The pick mortality rate in this study, which is 12.1 per 100 child-years, was observed in the first 6 months of follow up. The possible reason for this may be the immune reconstitution inflammatory syndrome (IRIS) which is in general abnormal immune response to antigens after the initiation of ART drugs mostly in the first 6 months of initiation.

The study indicates that, children who had severe anemia had higher hazard of mortality than those who had no severe anemia which was in line with studies conducted in Gondar, Tanzania and Thailand [12, 15, 23]. The possible justification could be, low hemoglobin level in anemic children leads to decreased oxygen delivery to cells which hinders the cells ability to maintain and repair its self. This condition will add burden on the TB/HIV co-infected child leading to mortality.

The extrapulmonary tuberculosis among TB/HIV co-infected children was four times more hazard of dying than those who are co-infected with pulmonary tuberculosis. Similar finding was reported by the study from Gondar comprehensive specialized hospital [12]. This may be due to the extrapulmonary forms of TB are mostly more severe than pulmonary TB. It may also affect multiple organs at the same time in the case of disseminated tuberculosis increasing the chance of death of the children who are already weakened by the HIV virus.

An additional factor that resulted from the analysis of this study was ART drug adherence. The children who had fair or poor drug adherence were 2.9 times more hazard of mortality than those with good ART drug adherence. This result is in line with a study conducted in India [24]. This may be because ART drugs have role in viral suppression which decreases the viral load helping the immune system to recover. Therefore, children with good adherence have better ability to fight the co-infection. But those who have fair or poor drug adherence may end up with treatment failure speeding up the viral replication thus leading to weakened immunity and death.

Furthermore, individuals co-infected with MDR TB and HIV had 5.7 times higher risk of mortality than children co-infected with non-drug resistant TB and HIV which is in line with the result of this study [23]. The reason for this can be, the treatment challenges that are experienced during treating the drug resistant strains that require second line regimens which have more adverse effects than the first line anti tuberculosis drugs affecting the co-infected child synergistically with the ART drugs. Moreover, as the treatment requires longer period than the non-drug resistant TB, it increases the children’s risk by exposing them for longer period.

### Limitation of the study

Since this study was conducted based on secondary data, charts excluded due to incomplete data and lost charts which may be related to mortality might underestimate the result of the study. The poor management of charts of children who died will also negatively affect the result. On the other hand, since TB is not recorded on most of the ART registers, it makes it difficult to find charts of all TB/HIV co-infected children on the study period which also affect the outcome. Lastly, the difficulties to diagnose tuberculosis among pediatrics will probably have effect on the result.

## Conclusion

Mortality rate of TB/HIV co-infected children in selected public hospitals in SNNPR, Ethiopia was documented as 2.78 per child years of observation as a result of this study. Moreover, Anemia, drug resistant tuberculosis, extrapulmonary TB and poor adherence to ART drugs were identified as the predictors of mortality among these children. The governmental and non- governmental organizations should provide trainings concerning screening, diagnosis and management of TB/HIV co-infection in children to health care providers. Prospective cohort study will be suggested on mortality among TB/HIV co-infected children in order to fill the gap of incomplete data.

## Data Availability

All relevant data are within the paper but any additional data required by the journal can be available anytime.

## Abbreviations

ABC: Abacavir
AHR: Adjusted Hazard Ratio
ART: Antiretroviral Therapy
AZT: Zidovudine
CD4: Cluster for Differentiation 4
CPT: Cotrimoxazole Prophylaxis Therapy
CHR: Crude Hazard Ratio
D4T: Stavudine
EFV: Efavirenz
Hgb: Hemoglobin
HIV: Human immune virus
IPT: Isoniazid Prophylaxis Therapy
IRIS: Immune reconstitution Inflammatory syndrome
MDR-TB: Multi-drug resistant tuberculosis
3TC: Lamivudine
NVP: Nevirapine
OIs: Opportunistic Infections
SDG: Sustainable developmental goal
SD: Standard Deviation
SNNPR: Southern Nation Nationalities and Peoples Region
TB: Tuberculosis
TDF: Tenofovir
UNAIDS: United Nations Program on HIV/AIDS
WAZ: Weight for age Z

## References

[1] Pan American Health Organization. TB/HIV coinfection regional clinical manual: 2017 Update [Internet]. 2018. 14–91 p. Available from: https://www.paho.org/en/node/58184

[2] Kliegman R, Stanton B, Schor N, St Geme J (2015). Nelson textbook of pediatrics - 20th edition (2 Vol set). Nelson Textbook of Pediatrics.

[3] European Centre for Disease Prevention and Control (2021). Annual Epidemiological Report for 2016 Key facts. Surveill Rep.

[4] UNAIDS and World Health Organization. Quality and Coverage of HIV Sentinel Surveillance With a brief History of the HIV / AIDS Epidemic. World Heal Organ UNAIDS. 2003;(September):8–13.

[5] Narendran G, Swaminathan S (2016). TB-HIV co-infection: a catastrophic comradeship. Oral Dis.

[6] World Health Organization (2015). Global Tuberculosis Report 2019, Advocacy Toolkit. Work Heal Saf.

[7] Turkova A, Chappell E, Judd A, Goodall RL, Welch SB, Foster C, et al. Prevalence, incidence, and associated risk factors of tuberculosis in children with HIV living in the UK and Ireland (CHIPS): a cohort study. Lancet HIV. 2015;2(12):e530–9. Epub 2015 Oct 29. PMID: 26614967. 10.1016/S2352-3018(15)00200-3 26614967

[8] Joint United Nations Programme on HIV and AIDS. Global HIV Statistics. 2020;(June):1–3. Available from: https://www.unaids.org/en/resources/fact-sheet

[9] Ethiopian Public Health Institute (2018). Ethiopia Population-Based HIV Impact Assessment (EPHIA)2017-2018. Summary Sheet: Preliminary Findings.

[10] WHO (2018). Roadmap towards ending TB in children and adolescents.

[11] Ethiopian Public Health Institute and Ministry of Health. Report on national TB/HIV sentinel surveillance (April 2010 - June 2015). Ethiop Public Heal Inst. 2015.

[12] Atalell KA, Tebeje NB, Ekubagewargies DT (2018). Survival and predictors of mortality among children co-infected with tuberculosis and human immunodeficiency virus at University of Gondar Comprehensive Specialized Hospital, Northwest Ethiopia. A retrospective follow-up study. PLoS One.

[13] Walters E, Duvenhage J, Draper HR, Hesseling AC, Van Wyk SS, Cotton MF (2014). Severe manifestations of extrapulmonary tuberculosis in HIV-infected children initiating antiretroviral therapy before 2 years of age. Arch Dis Child.

[14] Ebonyi AO, Oguche S, Agbaji OO, Sagay AS, Okonkwo PI, Idoko JA (2016). Mortality among pulmonary tuberculosis and HIV-1 co-infected nigerian children being treated for pulmonary tuberculosis and on antiretroviral therapy: a retrospective cohort study. Germs..

[15] Mwiru RS, Spiegelman D, Duggan C, Seage GR, Semu H, Chalamilla G, et al. Nutritional status and other baseline predictors of mortality among HIV-infected children initiating antiretroviral therapy in Tanzania. J Int Assoc Provid AIDS Care. 2015;14(2):172–9. Epub 2013 Oct 8. PMID: 24106055; PMCID: PMC4627587. 10.1177/2325957413500852 PMC4627587 24106055

[16] Adejumo OA, Daniel OJ, Adebayo BI, Adejumo EN, Jaiyesimi EO, Akang G, et al. Treatment outcomes of childhood TB in lagos, Nigeria. J Trop Pediatr. 2016;62(2):131–8. PMID: 26705331; PMCID: PMC4886120. 10.1093/tropej/fmv089 PMC4886120 26705331

[17] Buck WC, Olson D, Kabue MM, Ahmed S, Nchama LK, Munthali A, et al. Risk factors for mortality in Malawian children with human immunodeficiency virus and tuberculosis co-infection. Int J Tuberc Lung Dis. 2013;17(11):1389–95. PMID: 24125439; PMCID: PMC5523939. 10.5588/ijtld.13.0030 PMC5523939 24125439

[18] Hicks RM, Padayatchi N, Shah NS, Wolf A, Werner L, et al. Malnutrition associated with unfavorable outcome and death among South Malnutrition associated with unfavorable outcome and death among South African MDR-TB and HIV co-infected children. Int J Tuberc Lung Dis. 2014;18(9):1074–83. PMID: 25189555. 10.5588/ijtld.14.0231 25189555

[19] Isaakidis P, Casas EC, Das M, Tseretopoulou X, Ntzani EE, Ford N. Treatment outcomes for HIV and MDR-TB co-infected adults and children: systematic review and meta-analysis. Int J Tuberc Lung Dis. 2015;19(8):969–78. PMID: 26162364. 10.5588/ijtld.15.0123 26162364

[20] Bakeera-Kitaka S, Conesa-Botella A, Dhabangi A, Maganda A, Kekitiinwa A, Colebunders R, et al. Tuberculosis in human immunodeficiency virus infected Ugandan children starting on antiretroviral therapy. Int J Tuberc Lung Dis. 2011;15(8):1082–6. PMID: 21740672; PMCID: PMC3325109. 10.5588/ijtld.10.0538 PMC3325109 21740672

[21] Marcy O, Tejiokem M, Msellati P, Truong Huu K, Do Chau V, Tran Ngoc D, et al. Mortality and its determinants in antiretroviral treatment-naive HIV-infected children with suspected tuberculosis: an observational cohort study. Lancet HIV. 2018;5(2):e87–95. PMID: 29174612. 10.1016/S2352-3018(17)30206-0 29174612

[22] Carlucci JG, Blevins Peratikos M, Kipp AM, Lindegren ML, Du QT, Renner L, et al. Tuberculosis treatment outcomes among HIV/TB-Coinfected children in the international epidemiology databases to evaluate AIDS (IeDEA) network. J Acquir Immune Defic Syndr. 2017;75(2):156–63. PMID: 28234689; PMCID: PMC5429189. 10.1097/QAI.0000000000001335 PMC5429189 28234689

[23] Salvadori N, Ngo-Giang-Huong N, Duclercq C, Kanjanavanit S, Ngampiyaskul C, Techakunakorn P, et al. Incidence of tuberculosis and associated mortality in a cohort of human immunodeficiency virus-infected children initiating antiretroviral therapy. J Pediatric Infect Dis Soc. 2017;6(2):161–7. PMID: 28204517; PMCID: PMC5907848. 10.1093/jpids/piw090 PMC5907848 28204517

[24] Isaakidis P, Cox HS, Varghese B, Montaldo C, da Silva E, Mansoor H, et al. Ambulatory multi-drug resistant tuberculosis treatment outcomes in a cohort of HIV-infected patients in a slum setting in Mumbai, India.PLoS One. 2011;6(12):e28066. PMID: 22145022; PMCID: PMC3228724. 10.1371/journal.pone.0028066 PMC3228724 22145022

[25] Mukuku O, Mutombo AM, Kakisingi CN, Musung JM, Wembonyama SO, Luboya ON. Tuberculosis and hiv co-infection in Congolese children: risk factors of death. Pan Afr Med J. 2019;33:326. PMID: 31692828; PMCID: PMC6815491. 10.11604/pamj.2019.33.326.18911 PMC6815491 31692828

